# Supplementary material for: Lasp1 Expression Is Implicated in Embryonic Development of Zebrafish
Source: Genes (Basel). 2022 Dec 22;14(1):35. doi: 10.3390/genes14010035 (PMC9858601; doi:10.3390/genes14010035)
Supplement: Supplementary file 1 [file genes-14-00035-s001.zip › genes-2050316-supplementary.pdf]

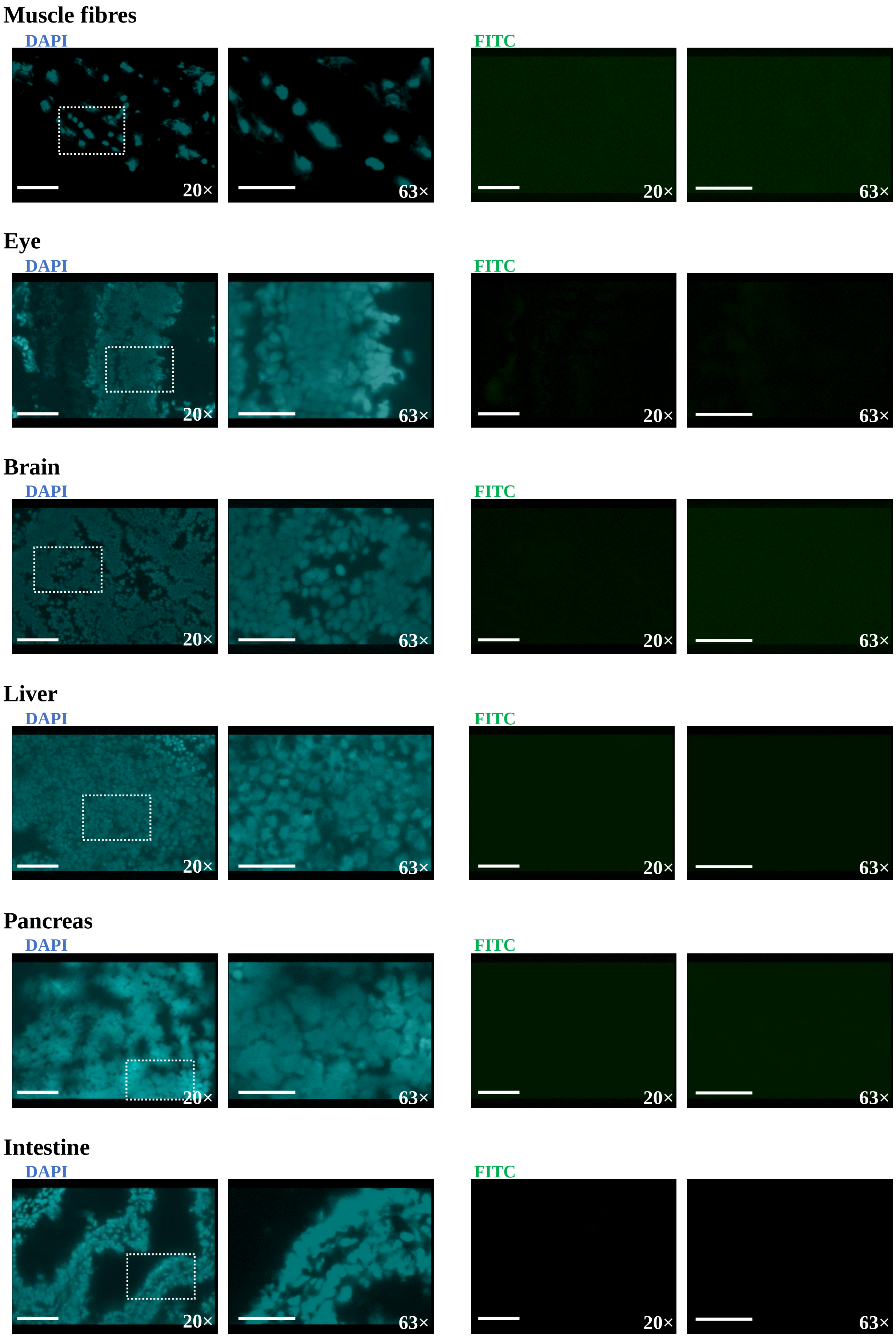

Figure S1. Negative control images for each selected tissue section of zebrafish. Magnification 20× and 63× (blue-DAPI, green-FITC). Scale bars are placed on the lower left corner of each image. They correspond to 100 µm for 20x magnification and to 30 µm for 63x magnification.

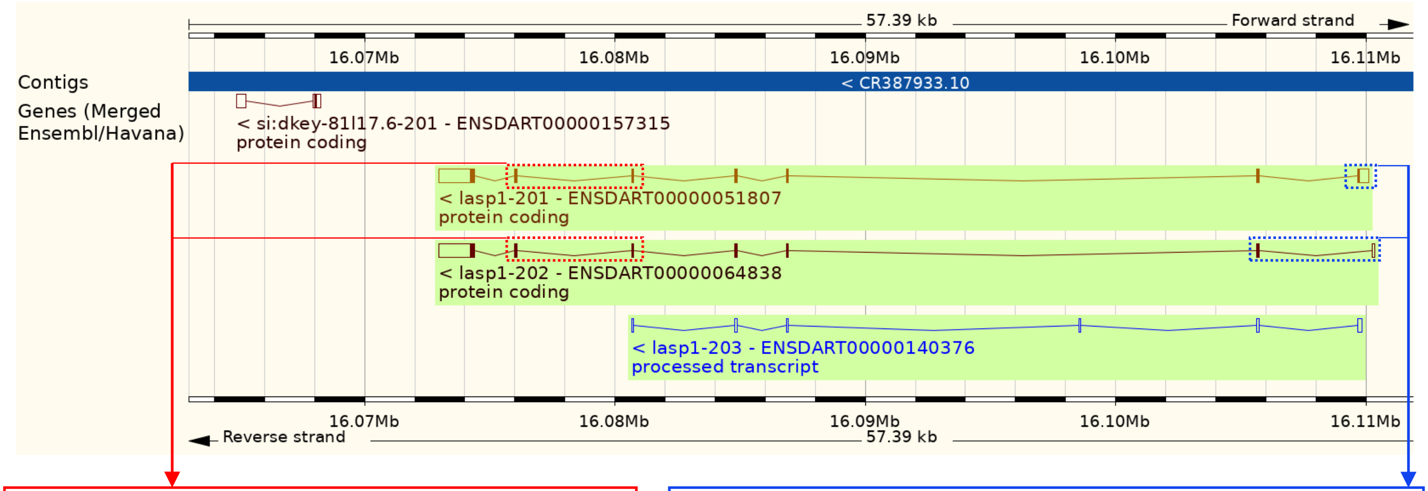

Location of the PCR probe assay

***lasp1-201* and *lasp1-202* - Exon 5**
Start: 16,080,744   End: 16,080,660   Length: 85
Sequence:
ATTAAATACCATGAGGACTTTGAGAAGAGCCGAAGTGGAGGGG
ACACCCCGCTCCCACTGACACCTCAACTAAACACACCTCCAG

***lasp1-201* and *lasp1-202* - Exon 6**
Start: 16,076,080   End: 16,075,992   Length: 89
Sequence:
CATATCCATCCTCGGCTGCATCCCAGAACTATCACTATGAGCC
TGAGCCGGTGCGTCCAGCGGCTGCAGCTCCTCCTCCCAGCTCT
GGG

Location of the sequences targeted by morpholinos

***lasp1-201* - Exon 1**
Start: 16,110,100   End: 16,109,658   Length: 443
Sequence:
ACTCCCAATATGAGTATCACAGTATCCGCTTGACGGTGTGATTTTTAAAA
CAAGTCAGCATTAAGTTTAACGCTTCCTAATACAAACTTTAACTAATTGA
ACTCAAAATCAGGACTTTTATCGATTTTAGATTGATATTAGATAGTGAAA
GTCTTCCGCGGAATGCCCTTGCTTATCTGTGCACGTGAGCGCGCAGCAGG
ACACAGCGGCAGAGCCGTGAGCGCGCCTTCCGCGTCTGCTTCGTTTCGCT
GACAGCCAGCGGTGCGAACTGGACTCATCAACGGCTACAGATCCACACCT
CAACAACACAACACTGCTTCTGCCGACCGAGTCCTCGTTTCATCCCTTCG
GCCTGCTGTTATTATTCCTCCAAAATGAACCCGCTGTGTAGCAGATGTAA
CCGAGTGGTGTATCCCACGGAAAAAGTGAATTGTCTTGACAAG

***lasp1-202* - Exon 1**
Start: 16,110,351   End: 16,110,263   Length: 89
Sequence:
AAACCAAACCAAAGCTCACGTTGAAGTATTTTTGGCGCATCCGATTCGA
AACTGACAATACTTTCATTTCATGCAGGAATGTGAATATG

***lasp1-202* - Exon 2**
Start: 16,105,731   End: 16,105,637   Length: 95
Sequence:
TACTGGCATAAAGGATGCTTTAGCTGTGAAGTCTGTAAGATGACTCTAA
ACATGAAGAACTACAAAGGCTTTGAGAAGAGACCATACTGTAATGC

Figure S2. Schematic representation of the location for PCR probe assay and morpholinos. In the upper scheme, zebrafish *lasp1* transcripts are reported, including *lasp-201*, *lasp-202*, and *lasp-203*. *lasp-201* and *lasp-202* encode a proteins of 234 and 212 ammino acids, respectively; *lasp-203* is indicated as non-protein coding RNA (Ensembl Release 108). The PCR probe assay (assay ID Dr03439091, Thermo Fisher Scientific) overlaps the region in exons 5 and 6 and it is specific for *lasp-201* and *lasp-202*; the corresponding sequence is underlined in red. To knock-down Lasp1, two specific morpholinos are used targeting the translation start site of *lasp-201* and of *lasp-202*. The morpholinos target sequences are underlined in blue. Sequence in orange corresponds to 5’UTR, the translated sequence is indicated in grey.

Protein Alignment of human LASP1 and zebrafish Lasp1

Sequence 1: NP_006139.1 **Gene:** *LASP1* / 3927 **HGNCID:** 6513 **Length:** 261 **Species:** Homo sapiens
Sequence 2: NP_997801.1 **Gene:** *lasp1* / 323216 **ZFINID:** ZDB-GENE-030131-1936 **Length:** 234 **Species:** Danio rerio

Alignment Length: 263 **Identity:** 179/263 - (68%)
Similarity: 198/263 - (75%) **Gaps:** 31/263 - (11%)

```
Human     1 MNPNCARCGKIVYPTEKVNCLDKFWHKACFHCETCKMTLNMKNYKGYEKKPYCNAHYPKQSFTMV 65
            |||.|:||.::||||||||||||:|||.||.||.||||||||||||:|:||||||||||.|||.|
Zfish     1 MNPLCSRCNRVVYPTEKVNCLDKYWHKGCFSCEVCKMTLNMKNYKGFEKRPYCNAHYPKTSFTSV 65

Human    66 ADTPENLRLKQQSELQSQVRYKEEFEKNKGKGFSVVADTPELQRIKKTQDQISNIKYHEEFEKSR 130
            |||||||||||||::||||.|||||||||||||||||||||||||||||||||||||||:|||||
Zfish    66 ADTPENLRLKQQSKMQSQVLYKEEFEKNKGKGFSVVADTPELQRIKKTQDQISNIKYHEDFEKSR 130

Human   131 MGPSGGEGMEPERRDSQDGSSYRRPLEQQ--QPHHIPTSAPVYQQPQQQPVAQSYGGYKEPAAPV 193
               |||:  .|            .||..|  .|...|:||          .:|:|....||..|.
Zfish   131 ---SGGD--TP------------LPLTPQLNTPPAYPSSA----------ASQNYHYEPEPVRPA 168

Human   194 SIQRSAPGGGGKRYRAVYDYSAADEDEVSFQDGDTIVNVQQIDDGWMYGTVERTGDTGMLPANYV 258
            :  .:.|...||||||||||:|||||||||.|||.||:|||||:|||||.|||||..||||||||
Zfish   169 A--AAPPPSSGKRYRAVYDYTAADEDEVSFMDGDMIVDVQQIDEGWMYGRVERTGQQGMLPANYV 231

Human   259 EAI 261
            ||:
Zfish   232 EAM 234
```

Domain	Region	Identity
LIM	5..57	41/51 (80%)
NEBU	62..92	25/29 (86%)
NEBU	98..128	28/29 (97%)
SH3	203..261	48/57 (84%)

Figure S3. Alignment of human LASP1 and zebrafish Lasp1 protein sequence using DIOPT (version 8). Sequences for each domain are in green. The identity and the region of Lasp1 domains are indicated in the table. LIM, Lin11-Isl1-Mec3 domain; NEBU, Nebulin-repeats; SH3, Src homology 3 domain.
